# Snapshots of ADP-ribose bound to Getah virus macro domain reveal an intriguing choreography

**DOI:** 10.1038/s41598-020-70870-w

**Published:** 2020-09-02

**Authors:** Ana Sofia Ferreira-Ramos, Gerlind Sulzenbacher, Bruno Canard, Bruno Coutard

**Affiliations:** 1 Architecture et Fonction des Macromolécules Biologiques, CNRS, Aix-Marseille Université, 13288 Marseille, France; 2 Unité des Virus Emergents (UVE), Aix Marseille Univ-IRD 190-INSERM 1207-IHU Méditerranée Infection, 13005 Marseille, France; 3 Present Address: Institute of Biochemistry, University of Lübeck, Ratzeburger Allee 160, 23562 Lübeck, Germany

**Keywords:** X-ray crystallography, Alphaviruses

## Abstract

Alphaviruses are (re-)emerging arboviruses of public health concern. The nsP3 gene product is one of the key players during viral replication. NsP3 comprises three domains: a macro domain, a zinc-binding domain and a hypervariable region. The macro domain is essential at both early and late stages of the replication cycle through ADP-ribose (ADPr) binding and de-ADP-ribosylation of host proteins. However, both its specific role and the precise molecular mechanism of de-ADP-ribosylation across specific viral families remains to be elucidated. Here we investigate by X-ray crystallography the mechanism of ADPr reactivity in the active site of Getah virus macro domain, which displays a peculiar substitution of one of the conserved residues in the catalytic loop. ADPr adopts distinct poses including a covalent bond between the C′′1 of the ADPr and a conserved *Togaviridae*-specific cysteine. These different poses observed for ADPr may represent snapshots of the de-ADP-ribosylation mechanism, highlighting residues to be further characterised.

## Introduction

Alphaviruses are arthropod-borne viruses among which several, such as Chikungunya virus (CHIKV) and Venezuelan equine encephalitis virus (VEEV), are emerging or re-emerging viruses. According to the 7th report of the International Committee on Taxonomy of Viruses (ICTV), the genus *Alphavirus*, belonging to the *Togaviridae* family, is organised into 31 species and at least ten of them are human pathogens^1^. Members of the *Alphavirus* genus are geographically and evolutionary distinguished into “Old World” (OW) alphaviruses, represented by CHIKV, and “New World” (NW) viruses, prototyped by VEEV, as well as several species corresponding to viruses that most likely appeared from recombination events between OW and NW viruses^2^ or viruses of marine origin.

Their genome is a positive-sense single-stranded RNA molecule which is usually 11 to 12 kilobases long. The genome carries two open reading frames (ORFs). The first ORF can be translated directly from the genomic RNA in P123, and P1234 polyproteins, which are sequentially processed into four non-structural proteins (nsP), named nsP1 to nsP4. The proteolysis intermediates and the final nsPs constitute the transcription/replication complex (TRC) organised in spherules at the plasma membrane. In this TRC, nsP3 has been for a long time the least understood nsP, but recent studies revealed some of its functions in viral replication^3^. nsP3 is organised into three domains: a macro domain (MD) at the N-terminal, a zinc-binding domain (ZBD) and a C-terminal hypervariable region (HVR). The HVR plays a key role in viral replication by interacting with several partners among which Ras GTPase-activating protein-binding protein (G3BP) and SH3 containing proteins^4^. The N-terminal domain is structurally associated to the class of macro domains, protein modules found in all domains of life. Despite structural conservation, macro domains participate in various cellular functions including DNA repair, cell differentiation, proliferation or apoptosis. Macro domains can recognize ADP-ribose (ADPr), poly-ADP-ribose (PAR) in their free or protein-bound form, or O-acetyl-ADP-ribose (OAADPr). In addition to binding, some macro domains can also catalyze several reactions such as de-ADP-ribosylation^5^. Like coronavirus or hepevirus macro domains^6,7^, alphavirus macro domains recognise ADP-ribose and have de-ADP-ribosylation activity. This latter functions are thought to be essential in both early and late replication steps^8^. It is postulated that de-mono-ADP-ribosylation is a viral countermeasure to cellular innate immunity mediated by the viral macro domain^9^.

The structures of the macro domains from alphavirus species VEEV, CHIKV, Sindbis virus (SINV), and Mayaro virus (MAYV) were determined using X-ray crystallography and/or NMR^10–12^. Structure and sequence analyses together with functional characterization allowed assignment of alphavirus macro domains to a protein family prototyped by human macroD2^7^. Viral macro domains were found originally to have ADR-ribose-1″-phosphate phosphatase (A1″Pase) activity^10,13^. More recently, it was shown that alphavirus macro domains are able to de-ADP-ribosylate Asp or Glu side-chains of host proteins^7,14,15^. Some of the residues involved in ADP-ribose binding and de-ADP-ribosylation have been characterized^9^. However, the molecular mechanism of de-ADP-ribosylation by alphavirus macro domain remains to be clarified.

**Figure 1:**
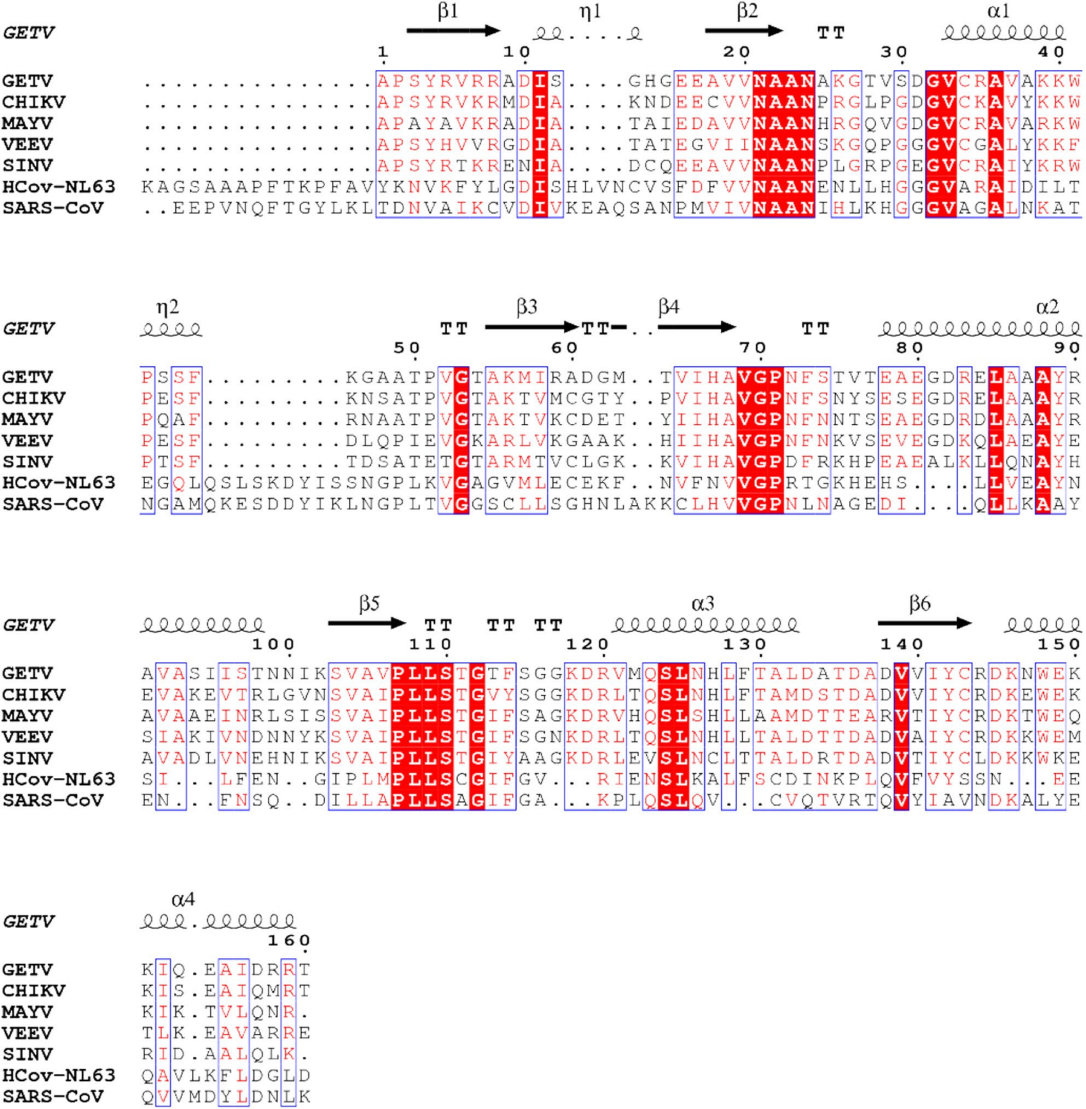
Sequence alignment of GETV macro domain with other viral macro domains. The OW group from the genus alphavirus is represented in the alignment by CHIKV, MAYV and SINV macro domains, while NW group is represented by the VEEV macro domain. The human coronavirus NL63 macro domain as well as SARS coronavirus are included. The alignment was obtained using ESPript and residues highlighted with red boxes are strictly conserved, red residues represent similarity in a group, and blue frames represent similarity across groups. Secondary structure elements derived from the GETV macro domain crystal structure are represented above the alignment.

In order to better understand this mechanism, we initiated a structure-based study of the Getah virus (GETV) macro domain, which shows a peculiar substitution for one of the most conserved residues in the catalytic loop, namely serine replacing a glycine in position 30 (Fig. 1). GETV is an alphavirus isolated for the first time in Malaysia in 1955 from *Culex *spp. mosquitoes. GETV is geographically distributed from Asia to the north of Australia and infects mainly horses^16^. We produced the GETV macro domain for crystallographic studies, alone or in complex with ADP-ribose, and documented several conformations adopted by ADP-ribose in the binding site. We show here that our structures provide snapshots that could contribute to unravel the still elusive de-ADP-ribosylation mechanism at play to de-activate innate immunity responses elicited by alphavirus infection.

Taken together, this ensemble of conformations might represent several snapshots of the de-ADP-ribosylation mechanism, highlighting residues that have not been evaluated to date.

## Materials and methods

### Protein production and purification

The DNA coding sequence of the nsP3 macro domain (amino-acids 1 to 160) of Getah virus (strain M1, GenBank: ABK32031.1, amino-acids 1,333 to 1,492) was optimized for expression in *Escherichia coli* and synthesized by THERMO FISCHER SCIENTIFIC. The coding sequence was cloned into a pDest14 vector using the “Gateway” cloning procedure (THERMO FISCHER SCIENTIFIC). A hexahistidine (6-His) coding sequence was added at the 3′ end to produce the GETV macro domain in fusion with a C-terminal 6-His tag. A short sequence of two codons coding for methionine and lysine were added to the 5′ end in order to allow the translation in *E. coli*. The bacterial growth conditions for optimal expression of GETV macro domain were obtained from an incomplete factorial expression screen^17^. GETV macro domain was produced in *E. coli* Rosetta (DE3) pLysS cells (NOVAGEN). Bacteria were grown at 37 °C with shaking (200 rpm) in Terrific Broth (TB) medium (SIGMA-ALDRICH) containing 34 µg·mL^−1^ chloramphenicol and 100 µg·mL^−1^ ampicillin. The expression of the recombinant gene was induced with 0.5 mM isopropyl β-D-1-thiogalactopyranoside (IPTG) when the bacterial cell density reached the exponential phase at OD_600nm_ of 0.4. After induction, the incubation temperature was dropped to 25 °C for overnight expression. Subsequently, the bacterial cells were harvested by centrifugation at 4,000 g for 15 min and pellets were resuspended in 50 mL of lysis buffer made of 50 mM TRIS–HCl pH 8.0, 300 mM NaCl, 10 mM imidazole, 5% glycerol, 0.1% Triton X-100 and one tablet of complete EDTA-free protease inhibitor cocktail (ROCHE). The resuspended pellets were stored at − 80 °C until the time of use. Pellets were thawed and 0.25 mg·mL^−1^ of lysozyme (SIGMA-ALDRICH), 10 µg·mL^−1^ of DNase I (SIGMA-ALDRICH) and 1 mM phenylmethylsulfonyl fluoride (PMSF) (SIGMA-ALDRICH) were added. The samples were incubated at 4 °C for 30 min and then sonicated. Samples were then centrifuged at 13,000 g for 1 h. The supernatant was loaded onto a 5 mL His prep column (GE HEALTHCARE) for Immobilized Metal Affinity Chromatography (IMAC) using an ÄKTA Xpress system (GE HEALTHCARE). After loading the supernatant, the column was washed with a buffer containing 50 mM TRIS-HCl, 300 mM NaCl, 50 mM imidazole, pH 8.0. The recombinant protein was eluted with 50 mM TRIS-HCl, 300 mM NaCl, and 250 mM imidazole, pH 8.0. Size exclusion chromatography was next performed on a Superdex 200 HiLoad 16/600 column (GE HEALTHCARE) equilibrated in 20 mM HEPES, 300 mM NaCl, pH 7.4. The eluted protein was concentrated up to 14 mg·mL^−1^ using an Ultracel regenerated cellulose membrane with a 3 kDa molecular weight cut-off (Amicon Ultra-15 Centrifugal Filter, MERCK MILLIPORE).

### Purity and stability assessment

The purity of the protein was assessed on SDS-PAGE gels stained with Coomassie blue. Besides, protein stabilization by ADP-ribose was additionality checked by thermal shift assay (TSA)^18^ in a 96-well thin-wall PCR plate (BIO-RAD) using a quantitative PCR machine ICycler IQ (BIO-RAD). Briefly, the protein at a final concentration of 0.3 mg·mL^−1^ was mixed with a SYPRO orange solution at concentrations recommended by the manufacturer (THERMO FISHER SCIENTIFIC) in a final volume of 25 µL and tested with the following concentrations of ADP-ribose: 0.5 mM, 0.25 mM, 0.125 mM, 0.063 mM, 0.031 mM, 0.016 mM and 0 mM. Accumulative steps of temperature from 20 to 90 °C were applied to the samples. The increase of the fluorescence emitted by the probe that binds the exposed hydrophobic regions of the denatured protein was used to monitor the denaturation of the protein. A melting temperature (*T*_*m*_) was calculated as the mid-log of the transition phase from the native to the denatured protein using a Boltzmann model (ORIGIN SOFTWARE).

### Crystallization, co-crystallization and crystal soaking

Initial crystallization trials were carried out with GETV macro domain at 14 mg·mL^−1^ using the commercial screens WIZARD CLASSIC 1&2 HT96, STRUCTURE 1&2 HT96, and STURA FOOTPRINT Screen 48 (MOLECULAR DIMENSIONS LIMITED) in 3-well sitting-drop SWISSCI crystallization plates (TPP LABTECH). 100 nL of crystallization solutions were added to 100, 200 or 300 nL of GETV macro domain solution using a MOSQUITO Robot (TTP LABTECH). The conditions where crystal hits were obtained were then optimized using either 96-well SWISSCI crystallization plates or 24-well hanging drop LINBRO plates. Co-crystallization experiments were set-up with GETV macro domain complemented with (1) ADP-ribose, (2) ADP-ribose and glutamic acid or (3) ADP-ribose and aspartic acid, with final concentrations of 3 mM for ADP-ribose, 50 mM for glutamic acid and 3 mM or 30 mM for aspartic acid. In general, crystals appeared after one day and were grown for approximately two weeks. Soaking experiments were performed with crystals of GETV macro domain co-crystallized with ADP-ribose by adding mother liquor supplemented with aspartic acid to the crystallization droplet followed by 2–3 h or overnight incubation at 20 °C. Afterwards, crystals were flash-cooled in liquid nitrogen using 20% of glycerol in the mother liquid as cryo-protectant.

### Data collection and structure determination

Diffraction intensities were recorded on beamlines ID23-1 and ID30A-3 at the European Synchrotron Radiation Facility (Grenoble, France) and on beamline Proxima-2 at Soleil Synchrotron (Gif-sur-Yvette, France). Indexing and integration of the different datasets were performed using MOSFLM^19^ or XDS^20^. Data were scaled and merged using the CCP4^21^ suite of programs POINTLESS^22^, AIMLESS ^23^ and TRUNCATE^24^. Random sets of approximately 5% of reflections, depending on the resolution limit, were set aside for FreeR cross-validation purposes. Where data-sets of ligand complexes were in the same space group as the native data set, the composition of cross-validation data sets was systematically taken over from the parent data set. The structure of native GETV macro domain was determined by molecular replacement with MolRep^25^ using the macro domain of Chikungunya virus (CHIKV, PDB 3GPG) as a search model. The structures of GETV macro domain in complex with ligands were determined either by molecular replacement using the native GETV macro domain as a search model or by difference Fourier synthesis.

Refinement was performed using REFMAC^26^, interspersed with cycles of manual model adjustments with COOT^27^. Ligands were fitted into unbiased Fo–Fc difference electron density maps calculated after 10 cycles of rigid-body refinement. Hydrogens were added in the riding position. Coordinates and restraints for ADP-ribose in the close conformation were retrieved from the CCP4 ligand dictionary and a model and restraints for ADP-ribose in the open conformation were generated with the CCP4 Monomer Library Sketcher. Model quality was assessed with internal modules of Coot and with the MOLPROBITY server^28^. Crystallographic models are of good quality with 99.4–100% of residues in favoured regions of the Ramachandran plot and no outliers. Data collection and refinement statistics are summarized in Table 1 with representative electron density depicted in Supplementary Fig. S1. Figures representing structural renderings were generated with the PyMOL MOLECULAR GRAPHICS SYSTEM (DeLano, W.L. The PyMOL Molecular Graphics on https://www.pymol.org/). Sequence alignments were made using Clustal Omega^29^ and graphical rendering of the alignments, considering structural information, were made with ESPript^30^. The atomic coordinates and structure factors have been deposited in the Protein Data Bank under accession numbers 6QZU (native GETV macro domain), 6R0F (GETV macro domain with ADP-ribose in close conformation pose 1), 6R0G, (GETV macro domain with ADP-ribose in close conformation pose 2), 6R0T (GETV macro domain with ADP-ribose in open ring conformation), 6R0P (GETV macro domain with ADP-ribose in double open ring conformation) and 6R0R (GETV macro domain with covalently bound ADP-ribose).

**Table 1:** Data collection and refinement statistics.

	Native	Closed ribose “pose 1”	Closed ribose “pose 2”	Open ribose	Open ribose in double conformation	ADP-ribose covalently bound to Cys34
**Data collection**						
Beam line	ESRF ID23-1	ESRF ID30A3	ESRF ID30A3	ESRF ID30A3	ESRF ID30A3	SOLEIL Proxima2
Space group	P2_1_2_1_2_1_	P2_1_2_1_2_1_	P2_1_2_1_2_1_	P2_1_2_1_2_1_	P2_1_2_1_2_1_	C2
**Cell dimensions**						
*a, b, c* (Å)	46.55, 71.36, 94.95	46.73, 71.57, 98.69	46.88, 71.65, 98.80	46.67, 71.44, 98.97	46.70, 71.45, 99.50	64.01, 46.80, 51.00; β = 104.16
Resolution (Å)	41.80–2.00 (2.05–2.00)	42.23–2.05 (2.11–2.05)	42.35–1.70 (1.73–1.70)	40.68–1.85 (1.89–1.85)	40.83–1.60 (1.63–1.60)	37.37–1.45 (1.47–1.45)
R_*merge*_	0.134 (0.748)	0.144 (1.328)	0.062 (1.012)	0.078 (1.221)	0.059 (1.268)	0.046 (0.826)
R_*pim*_	0.079 (0.448)	0.073 (0.660)	0.040 (0.664)	0.039 (0.591)	0.034 (0.710)	0.019 (0.484)
CC(1/2)	0.989 (0.603)	0.994 (0.468)	0.999 (0.527)	0.998 (0.627)	0.998 (0.559)	0.999 (0.352)
*I*/σ*I*	7.3 (2.1)	8.5 (1.5)	15.6 (1.6)	13.1 (1.40)	13.1 (1.2)	20.6 (2.1)
Completeness (%)	99.8 (99.8)	98.6 (99.0)	97.5 (97.4)	99.6 (99.8)	98.7 (97.5)	99.9 (100)
Redundancy	4.5 (4.3)	4.8 (5.1)	5.0 (5.3)	4.8 (5.1)	4.0 (4.1)	6.6 (6.5)
Wilson B (Å^2^)	13.3	25.50	18.9	18.34	15.36	17.39
**Refinement**						
Resolution (Å)	57.05–2.00	40.66–2.05	40.70–1.70	33.98–1.85	36.41–1.60	37.40–1.45
No. reflections	20,700	19,838	34,198	27,122	41,597	24,542
R_*work*_	19.39 (27.70)	19.27 (31.6)	18.11 (34.20)	16.84 (30.90)	16.94 (48.50)	16.95 (53.20)
R_*free*_	23.11 (28.70)	23.55 (32.50)	20.75 (33.50)	19.90 (34.00)	19.35 (51.70)	19.67 (54.60)
*No. atoms*						
Protein	2,386	2,386	2,388	2,388	2,388	1,210
ADP-ribose	–	72	72	72	72	36
Water/ligands	190/11	184/-	231/8	190/24	279/8	185/4
*B-factors (Å*^*2*^*)*						
Protein	12.11	37.98	27.57	46.05	26.41	19.81
ADP-ribose	–	49.11	29.20	48.32	27.06	19.61
Water/ligands	34.72/47.06	37.03/-	33.42/40.84	50.42/59.22	34.40/27.28	29.13/29.04
*R.m.s. deviations*						
Bond lengths (Å)	0.009	0.007	0.005	0.007	0.006	0.009
Bond angles (°)	1.304	1.436	1.250	1.454	1.446	1.583
Ramachandran favoured	99.68	99.68	100	99.68	99.68	99.38
PDB ID	6QZU	6R0F	6R0G	6R0T	6R0P	6R0R

Values in parentheses are for the highest-resolution shell.

## Results and discussion

### Protein production, crystallization and structure determination

**Figure 2:**
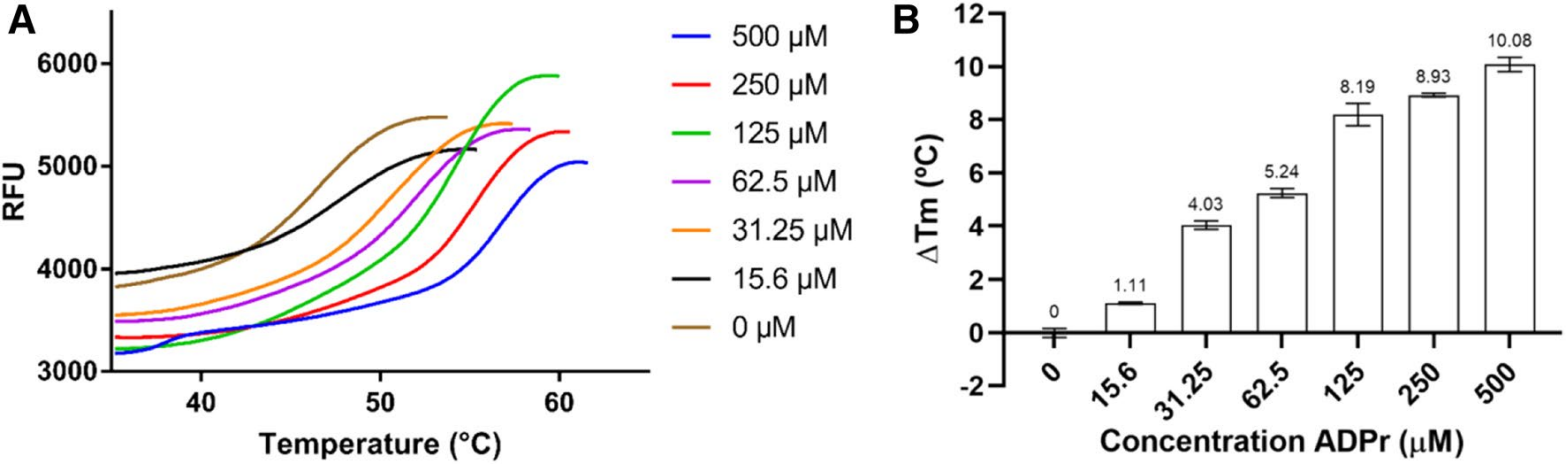
Effect of ADP-ribose concentrations on the thermostability of GETV macro domain. (**A**) Titration of ADP-ribose using the thermal denaturation shift assay on GETV macro domain. For each concentration of ADP-ribose titrated onto the GETV macro domain the melting temperature was calculated (T_m_). ΔTm [(Tm at a given concentration of ADP-ribose) minus (Tm with no ADP-ribose)] was calculated to quantify the change in protein stability (**B**).

The sequence corresponding to the putative macro domain on the GETV nsp1234 sequence (GenBank reference ABK32031.1) is encompassing aa 1,333 to 1,492 (positions in the polyprotein; hereafter numbered 1–160 for convenience). Its sequence identity with alphavirus homologues with known structures such as CHIKV and VEEV macro domains is 69% and 57%, respectively (Fig. 1). Macro domains harbour the consensus motif G(D/E/G)GV^7^. A focused analysis of these residues (amino acids 30 to 33 of the nsP3 sequence) of macro domains revealed that the consensus motif is followed by a *Togaviridae*-specific cysteine. It can also be noticed that the GETV macro domain is an exception regarding the consensus motif, as its sequence harbours a serine instead of a glycine at position 30. We thus first verified that this macro domain is a *bona fide* ADP-ribose binding module. The recombinant GETV macro domain was produced in *E. coli* and purified under non-denaturing conditions. After purification, the integrity of the protein was assessed by thermal shift assay (TSA). TSA experiments showed that the protein could be denatured by heat in a classical folded-to-denatured transition phase (data not shown) with a melting temperature (*T*_*m*_) of 46.5 ± 0.2 °C. Addition of 0.5 mM ADP-ribose to the protein solution led to a 10 °C increase of the *T*_*m*_ suggesting that the recombinant GETV macro domain indeed binds ADP-ribose (Fig. 2).

### Structure of the GETV macro domain and comparison with other (alphavirus) macro domains

The structure of the GETV macro domain was determined at 2.0 Å resolution by molecular replacement using the CHIKV macro domain (3GPG) as a template. The crystals of the GETV macro domain belong to the space group P2_1_2_1_2_1_ and two molecules are present in the asymmetric unit. All residues from Ala1 to Thr160 are well defined in the electron density and the model has excellent stereochemistry with 99.2% of side-chain rotamers in the favoured conformation and 99.7% of residues in the favoured Ramachandran plot regions (Table 1).

**Figure 3:**
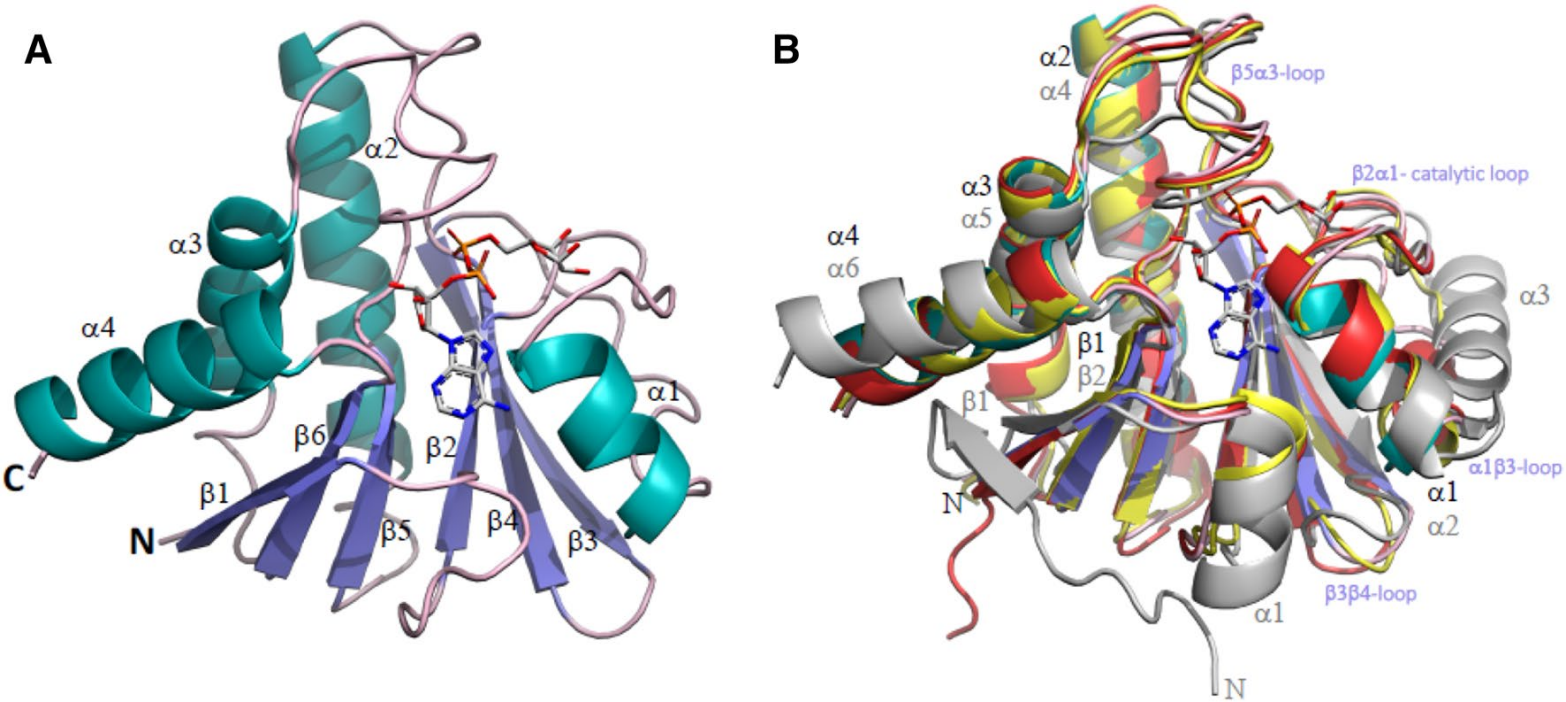
Overall structure of GETV macro domain. (**A**) Cartoon representation of GETV macro domain with APD-ribose depicted as seen in the GETV macro domain ADP-ribose complex “pose 1”. β-sheets are coloured in slate, α-helices in teal and loops in pink. Secondary structure elements and the N- and C-termini are labelled. ADP-ribose is represented in stick-mode, with carbon atoms coloured in grey, oxygens in red, nitrogen atoms in blue and phosphorus atoms in orange. (**B**) Overlap of GETV macro domain, same colour coding as in (**A**), with the structures of CHIKV macro domain (3GPO) in red, VEEV macro domain (3GQ) in yellow and the SARS macro domain (2FAV) in grey. The N-termini, important loops and α-helices are labelled in black and violet for the GETV macro domain and in grey for SARS macro domain. For clarity, only the first β-strand is labelled. APD-ribose as observed in the GETV macro domain ADP-ribose complex “pose 1” and colour-coded as in (**A**) has been added for reference.

The structure of GETV macro domain consists of a central twisted six-stranded β sheet (strand order β1, β6, β5, β2, β4, β3) sandwiched between one α-helix (α1) at one side and three α-helices (α2, α3 and α4) at the opposite site (Fig. 3A). The two molecules present in the asymmetric unit are virtually identical, with an r.m.s.d. of 0.22 Å between the two chains. A structural homology search with the DALI server^31^ revealed closest homology with macro domains from the alphavirus family such as CHIKV (3GPG), SINV (4GUA), VEEV (3GQO) and MAYV (5IQ5) with Z-scores in the range of 32.2 to 27.8 and r.m.s.d. values of 0.7 to 1.4 Å for 158–160 pairwise aligned C^α^ positions.

A second group of close structural homologues, with Z-scores in the range of 21.4 to 19.0 and r.m.s.d values of 1.8 to 2.2 Å for 151–154 pairwise aligned C^α^ positions comprises the structures of human macro domains (histone macroH2A1.1 [1ZR3], ARTD8 macrodomains 1 and 2 [3VFQ], MacroD2 [4IQY]), and a putative phosphatase (1SPV) and YmdB (5CB5), a O-acetyl-ADP-ribose deacetylase, from *E. coli*. Interestingly, lower structural homologies than for the above structures of human and bacterial macro domains are calculated by DALI for the viral macro domain structures from the coronavirus family (SARS-CoV [2FAV], Human-CoV NL63, [2VRI], Human-CoV 229E [3EJG], MERS-CoV [5DUS], feline-CoV [3ETI], Avian infectious bronchitis virus (IBV) strains Beaudette [3EKE] and M41 [3EWO]), with Z-scores in the range of 16.4 to 18.8 and r.m.s.d values of 1.8 to 2.3 Å for 136–143 pairwise aligned C^α^ positions. Thus structure-based phylogeny of macro domain does not cluster viral alphavirus and coronavirus together, raising questions on possible functional variations.

A comparison of the overall structure of GETV macro domain with the structures of macro domains from other alphaviruses shows that the alphavirus macro domain fold is very well conserved. In particular, the C^α^ chains of macro domains from the three Old World alphaviruses GETV, CHIKV and SINV superimpose well, as illustrated by the low root mean square deviation (r.m.s.d.) values of approximately 0.7 Å. Only minor structural changes are observed at the level of the β3β4 loop (Fig. 3B). The structural homology of the GETV macro domain (OW) with other alphavirus macro domains such as VEEV macro domain (NW) and the MAYV macro domain (OW) was slightly lower with r.m.s.d. values of 0.9 and 1.4 Å, respectively. Here the main structural differences occur in the loops branching structural elements (1) α1 and β3, (2) β3 and β4 and (3) β5 and α3. The β2 α1 loop, referred to as the catalytic loop, is structurally well conserved among alphavirus macro domain structures. With respect to the closest human or coronavirus macro domain structures, chiefly consisting of a central seven-stranded β-sheet flanked on either sides by three α-helices, alphavirus macro domains lack the first β-strand, and α-helices α1 and α3 (SARS-CoV annotation) are degenerated to 3_10_-helices or reduced to loops.

### Structures of the GETV macro domain in complex with ADP-ribose

The early evaluation of the integrity of GETV macro domain by monitoring the thermostability using thermal shift assay revealed an increase of *T*_*m*_ by 10 °C induced by the presence of small amounts of ADP-ribose, advocating that similar to other alphavirus macro domains, GETV macro domain can bind ADP-ribose. Therefore, we produced crystals of the GETV macro domain in the presence of 3 mM ADP-ribose. The study of the structure of the di-manganese mono-ADP-ribosylhydrolase DraG with a trapped amino acid-ADP-ribose reaction intermediate, Lys-ADP-ribose^32^, inspired us to add glutamic or aspartic acids to the crystallization trials, based on the rational that in this way we could reverse the de-ADP-ribosylation reaction mediated by alphavirus macro domain, documented to de-ADP-ribosylate carboxylic amino-acid side-chains^7,14,15^. Thus, we added glutamic or aspartic acids to co-crystallization or soaking solutions at equal or five to fifteen-times higher concentrations than that of ADP-ribose. All the optimal crystallization and co-crystallization solutions converged to the following buffer composition: 0.2 M imidazole/malate pH 5.9 ± 0.2 and 34 ± 4% of PEG 4 K. The crystallization and soaking procedures that led to the structures discussed in this study are summarize in Table 2, and data collection and refinement statistics are presented in Table 1. No clear electron density could be detected for aspartic or glutamic acid in any of the crystal structures. However, structural analyses revealed that the addition of the amino acids to the crystallization medium was associated with conformational changes both in the catalytic loop and of ADP-ribose. Five different conformations could be observed for ADP-ribose bound to the GETV macro domain, documented here below.

**Table 2:** Summary of crystallization procedures.

PDB code	Method/component	Crystallization solution	Conformation of ADP-ribose
6QZU	Crystallization GETV macro domain 16 mg·mL^−1^	0.2 M imidazole/malate pH 6.0, 30% PEG 4 K	
6R0F	Co-crystallization GETV macro domain 13 mg·mL^−1^, 3 mM ADP-ribose Soacking 15 mM aspartic acid	0.2 M imidazole/malate pH 5.9, 34% PEG 4 K	ADP-ribose with closed distal ribose, “pose 1”
6R0G	Co-crystallization GETV macro domain 13 mg·mL^−1^, 3 mM ADP-ribose, 50 mM glutamic acid	0.2 M imidazole/malate pH 6.0, 30% PEG 4 K	ADP-ribose with closed distal ribose, “pose 2”
6R0T	Co-crystallization GETV macro domain 13 mg·mL^−1^, 3 mM ADP-ribose + 30 mM aspartic acid	0.2 M imidazole/malate pH 5.9, 34% PEG 4 K	ADP-ribose with open distal ribose
6R0P	Co-crystallization GETV macro domain 13 mg·mL^−1^, 3 mM ADP-ribose, 30 mM aspartic acid	0.2 M imidazole/malate pH 6.0, 32% PEG 4 K	ADP-ribose with open distal ribose in double conformation
6R0R	Co-crystallization GETV macro domain 13 mg·mL^−1^, 3 mM ADP-ribose, 3 mM aspartic acid	0.2 M imidazole/malate pH 5.9, 38% PEG 4 K	ADP-ribose with distal ribose covalently linked to Cys34

### Structure of the GETV macro domain in complex with ADP-ribose presenting the distal ribose in “pose 1”

**Figure 4:**
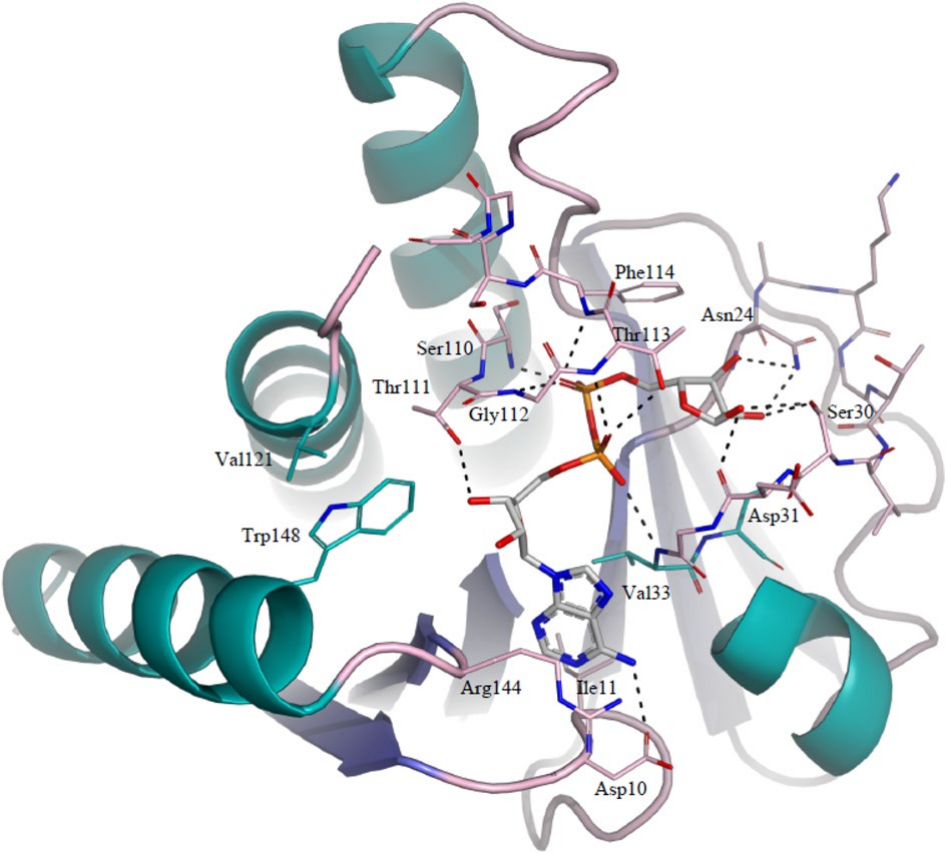
Interaction network between GETV macro domain and ADP-ribose in “pose 1”. ADP-ribose and interacting GETV macro domain residues are depicted in sticks and colour-coded as in Fig. 3A.

**Figure 5:**
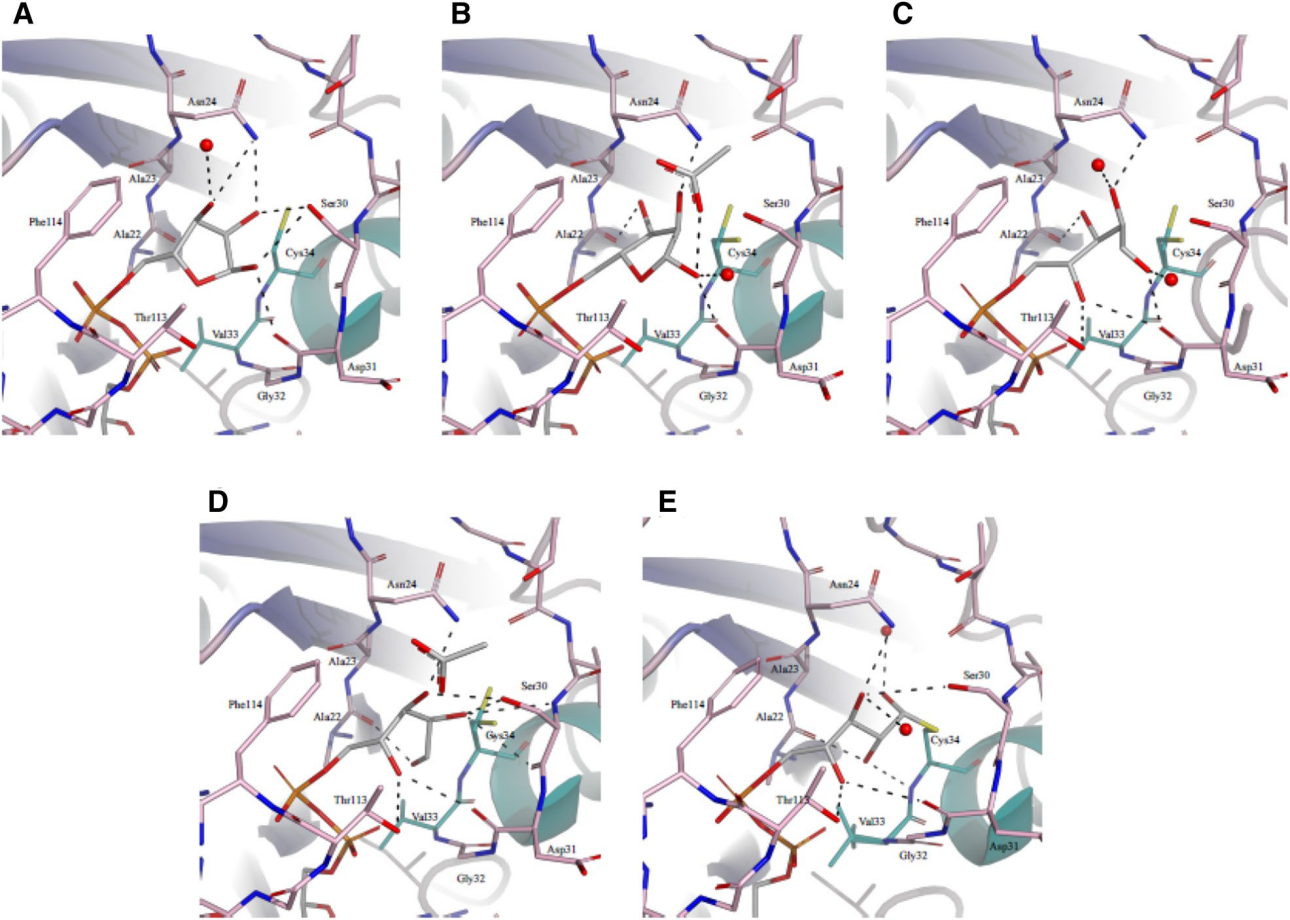
The conformational trajectory of ADP-ribose bound to GETV macro domain, triggered by the presence of aspartic or glutamic acid. The overall structure of GETV macro domain is shown in cartoon and interacting residues and ADP-ribose are depicted in sticks and colour-coded as in Fig. 3A. Hydrogen bonds are shown in dashed lines. (**A**) Complex with ADP-ribose in “pose 1”. (**B**) Complex with ADP-ribose in “pose 2”. (**C**) Complex with ADP-ribose in the open conformation (conformation A of complex with ADP-ribose in double open conformation). (**D**) Complex with ADP-ribose in the single open conformation. (**E**) Complex with ADP-ribose covalently bound to Cys34.

In a first instance we obtained diffraction data extending to 2.05 Å resolution for GETV macro domain in complex with ADP-ribose from a crystal obtained by co-crystallization of GETV macro domain with 3 mM ADP-ribose and subsequently soaked in mother-liquor solution supplemented with 15 mM aspartic acid. The structure was solved by difference Fourier synthesis and, as for the native structure, all residues are well defined in the electron density and the final model has excellent stereochemistry. Clear difference electron density could be observed for ADP-ribose bound to each of the two macro domain chains present in the asymmetric unit. GETV macro domain binds ADP-ribose in a deep cleft and the distal ribose ring adopts a pose as observed in most other macro domain ADP-ribose complexes, henceforth called “pose 1”. The adenine moiety binds via the N-1 atom to the main-chain of Ile11 and via the N-6 atom to the side-chain of Asp10, and makes stacking interactions at one side with the side-chains of Ile11 and Val33, and at the other side with the side-chain of Arg144 (Figs. 4, 5A). The interaction of N-6 with aspartate residues structurally equivalent to Asp10 is well conserved in most macro domain/ADP-ribose complexes and has been shown to be crucial for ADP-ribose binding in the *Archaeoglobus fulgidus* macro domain^33^. At the other hand, Arg144 is not conserved, not even within the alphavirus macro domain family, and its side-chain is rather disordered in all the structures described in this work, indicating that the stacking interaction with the adenine ring makes probably only weak contributions to the binding energy. The proximal ribose interacts with GETV macro domain through a single hydrogen bond between 3′-OH and the side-chain of Thr111. It is noteworthy that Trp148, conserved in alphavirus macro domains, protrudes into the ADP-ribose binding site and makes a steric clash with the 3′-OH. In the other alphavirus macro domain structures, the side-chains of the equivalent Trp residues are shifted about 1.5 to 2 Å away from the ADP-ribose binding site with respect to the position of Trp148 in the GETV macro domain. Here, a Val residue pointing from the back towards the Trp148 indole ring, Val121, impedes an equivalent rearrangement of the Trp148 side-chain, which appears to be highly dynamic and could not be modelled in a satisfactory fashion in all the structures described in this work.

Similarly to what had been observed in other macro domain/ADP-ribose complexes, the phosphate groups of ADP-ribose are lined by the catalytic loop β2α1 and loop β5α3 and tightly coordinated by hydrogen bonds with main-chain atoms of residues Val33, Ser110, Gly112, Thr113 and Phe114 and the side-chain of Thr113. Finally, the distal ribose, observed in the anomeric α-configuration, interacts with GETV macro domain through hydrogen bonds contracted between 3″-OH and the side-chain of Asn24, between 2″-OH and the side-chains of Asn24 and Ser30, and between 1″-OH and the side-chain Ser30 and the main-chain of Asp31, and establishes a stacking interaction with the side-chain of Phe114. The Ser30 of the catalytic β2α1 loop is unique to GETV macro domain and substituted by Gly in other viral macro domains harbouring the glycine-rich motif _30_G(D/E/G)GV_33_. The tight interactions established between the distal ribose and Ser30 point towards a role in substrate binding or catalysis for this residue. Not surprisingly, we found that the most important structural changes occurring in GETV macro domain upon ADP-ribose binding could be observed in the catalytic loop β2α1 and loop β5α3, which close-up over the ligand to establish tight binding interactions.

### Structure of the GETV macro domain in complex with ADP-ribose presenting the distal ribose in an unusual conformation, “pose 2”

By adding 3 mM ADP-ribose and 50 mM glutamic acid to the crystallization medium of GETV macro domain we obtained a second crystal for a GETV macro domain/ADP-ribose complex for which diffraction data extending to 1.7 Å resolution were collected. The structure was determined by difference Fourier synthesis and the final model has excellent stereochemistry. Thr160 in chain A could not be modelled in satisfactory fashion and in chain B electron density revealed the presence of Lys0, originating from the cloning strategy that includes an AAA triplet (Lys codon) to promote efficient translation initiation^34^. In this second GETV macro domain/ADP-ribose structure the interactions between the GETV macro domain and the adenine moiety, the proximal ribose and the phosphate groups are virtually identically to the interactions described above. However, the distal ribose is tilted by approximately 60° with respect to the “classical” pose described above and observed in all other macro domain/ADP-ribose complexes described so far (Fig. 5B), with exception of the crystal structure of human histone macroH2A1.1 in complex with ADP-ribose in form B (3IIF)^35^. However, in this latter crystal structure the distal ribose is tilted in the opposite direction with respect to the distal ribose observed in the present GETV/ADP-ribose structure. In the novel and unusual pose 2 the distal ribose, here in the b-anomeric configuration, establishes hydrogen bonds with Ala22 and Asn24 main-chain atoms via 3″-OH, with the side-chains of Asn24 and Ser30 via 2″-OH and with the Ser30 side-chain and the Asp31 main-chain mediated by the 1″-OH hydroxyl group. The ribose ring lines up in perfectly parallel fashion with the side-chain of Phe114. Interestingly, residual difference electron density was observed close to the distal ribose in chain A and was modelled as an acetate molecule, assuming that it represented the ordered part of glutamic acid added to the crystallization medium. This carboxylic moiety coordinates the 1″-OH and 2″-OH hydroxyl groups of ADP-ribose and is further stabilized by an H-bond interaction with Ser30. We dare to speculate that the position of the acetate molecule relative to the distal ribose ring is reminiscent of a putative ADP-ribose-glutamate conjugate, a possible substrate for GETV macro domain as inferred from the function of other alphavirus macro domains^7,14,15^. And yet again, Ser30 appears to play a crucial role in substrate binding.

### Structure of the GETV macro domain in complex with ADP-ribose presenting the distal ribose in an open ring conformation

We further obtained two additional GETV macro domain/ADP-ribose complexes by co-crystallizing the protein in the presence of 3 mM ADP-ribose and 30 mM aspartic acid. Diffraction data were recorded to 1.85 and 1.6 Å, respectively. Again, in these two crystal structures the interactions between GETV macro domain and the adenine moiety, the proximal ribose and the phosphate groups are comparable to those seen in the above-described models. Conversely, preliminary electron density maps, calculated before the incorporation of the ligand, clearly indicated that the distal ribose was present in the open conformation (Supplementary Fig. S1). In the complex structure at resolution of 1.85 Å the distal ribose is present in a single conformation, whereas the best way to account for difference electron density in the 1.6 Å data set was to model the distal ADP-ribose in a double open conformation (Fig. 5C,D). Both structures were determined by difference Fourier synthesis and the final models present excellent stereochemistry. In both structures no clear electron density could be observed for Thr160 in chains A and the additional Lys0 is present in chains B. In one of the conformations of ADP-ribose in the double open conformation, conformation A (Fig. 5C), the carbon atoms of the distal ribose superpose with the carbon atoms of the distal ribose ring as seen in the complex with ADP-ribose in pose 2 and the hydrogen-bonding interactions are to a large extent comparable. However, due to the ring opening the 4′′-OH is now free to make hydrogen-bonding interactions with the side-chain of Thr113 and the main-chain of Asp31. The acetate molecule, as observed in the complex with ADP-ribose in pose 2, could not be spotted in this structure. The conformations of the distal ribose in conformation B and the one in the complex with open ADP-ribose in single conformation are virtually identical (Fig. 5D). Here the linear ribose chain underwent a ~ 100° rotation around the bond connecting carbon atoms 4″-C and 3″-C, and consequently the hydrogen-bonding pattern between ADP-ribose and the GETV macro domain changed drastically. In this new conformation the 4″-OH binds the side-chain of Thr113 and the main-chain of Asp31, 3″-OH interacts with the side-chains of Asn24 and Ser30, the 2″-OH hydroxyl group contacts the side-chains of Ser30 and Cys and 1″-OH is coordinated by the main-chain atoms of Ala22 and Cys34 and hydrogen bonds the α-phosphate. An acetate molecule, reminiscent of aspartic acid added to the crystallization solution, is isosteric to the one observed in the complex with the distal ribose in pose 2 and interacts with the 3″-OH hydroxyl of ADP-ribose and the side-chain of Ser30.

### Structure of the GETV macro domain/ADP-ribose complex with the distal ribose covalently bound to Cys34

Finally, we obtained a last GETV macro domain/ADP-ribose complex by co-crystallizing the protein with 3 mM ADP-ribose and 3 mM aspartic acid and diffraction data were collected to 1.45 Å resolution. For no apparent reason the space group in this complex changed from P2_1_2_1_2_1_ (as observed in all the previously described structures) to C2 and only one molecule is present in the asymmetric unit. We verified cautiously whether the position of ADP-ribose in this novel complex could have influenced the crystal packing and could not retrieve any plausible explanation for the change of space group. The structure was solved by molecular replacement, using the native GETV macro domain structure as a search model and the final model presents very good stereochemistry. The interactions between GETV macro domain and the adenine moiety, the proximal ribose and the phosphate groups of ADP-ribose are specular to the interactions observed in all the GETV macro domain/ADP-ribose complexes described so far. To our surprise, ADP-ribose in this structure is found in the open conformation and a covalent bond is established between 1″-C and Cys34 SG (Fig. 5E and Sup. Fig. S1). It is noteworthy that in all the above described GETV macro domain/ADP-ribose structures the residues of the catalytic loop β2α1 and of the loop β5α3 are isosteric. The only exception is represented by Cys34, which adopts a double conformation in the complexes with ADP-ribose in pose 2 and ADP-ribose in the open conformation, with one of the alternate conformations of Cys34 pointing towards the distal ribose (Fig. 5B–D). Conversely, in the structure of the ADP-ribose covalent adduct a movement of approximately 1 Å could be observed for residues Ser30-Val33 of the catalytic loop β2α1. This structural rearrangement of the catalytic loop is conceivably due to a positional shift of the distal ribose chain with respect to open ribose in the single open conformation, implying a rotation of the of about 100° around the 3″-C and 2″-C bond. In the covalent complex the hydrogen bonds between 4″-OH and the side-chain of Thr113 and the main-chain of Asp31, as well as the one between 3″-OH and the side-chain of Asn24 are still maintained. However the interaction between 3″-OH and the side-chain of Ser30 is lost, and therefore the interaction between ADP-ribose and GETV macro domain is loosened. Due to the rearrangement of the open ADP-ribose chain the 2″-OH hydroxyl group now interacts with the main-chain of Cys34 and the 1″-OH group coordinates a water molecule.

## Discussion

Macro domains, named after the non-histone domain of histone variant macroH2A1.1, in which a macro domain was originally characterized^36^, are found in all kingdoms of life and have been shown to bind ADP-ribose and metabolites thereof. Although all sharing a common structural scaffold, they have diverged throughout evolution and some members support divers enzymatic activities such as O-acetyl-ADP-ribose deacetylation, ADP-ribose 1′′-phosphate dephosphorylation, as well as hydrolytic removal of mono- and poly-ADP-ribose attached to proteins. Diversification of enzymatic activities and substrate specificity goes necessarily along with variations in catalytic scenarios and different catalytic mechanisms have been proposed for macro domains, all converging on the central role of the catalytic β2α1 loop. Whereas acid-base catalysis occurring via an oxocarbenium intermediate and carried out by a glutamate residue has been proposed for *Thermomonospora curvata* PAR glycohydrolase PARG^37^, the human terminal ADP-ribose protein glycohydrolase TARG1 employs a lysine residue for nucleophilic attack at the C1′’ position of ribose attached to the acceptor protein, thereby forming a transient Schiff base intermediate further resolved by an aspartic acid^38^. Finally, a substrate-assisted mechanism for cleavage of the ADP-ribose/protein linkage had been proposed for human MacroD2 where the α-phosphate of the conformationally strained ADP-ribose activates an ideally positioned water molecule for nucleophilic attack on the C1′′ atom^39^. Seemingly the ubiquitous macro domains are built on a common globular scaffold prone to accommodate ADP-ribose. Still, subtle variations in loop structures elicit profound mechanistic diversity, which calls for further functional and structural dissection of this interesting class of molecules.

Intrigued by the substitution of a highly conserved glycine residue for serine and the presence of a *Togaviridae*-specific cysteine in the catalytic loop of GETV macro domain, we set out for a comprehensive structural investigation deemed to unravel the functional role of these substitutions. Inspired by the structural study of the mono-ADP-ribosylhydrolase DraG in which an amino-acid/ADP-ribose intermediate was trapped^32^, we co-crystallized or soaked GETV macro domains with ADP-ribose and with increasing concentrations of aspartic or glutamic acid, which could mimic host protein ADP-ribosylated side-chains, thereby reversing the de-ADP-ribosylation reaction. In a first ADP-ribose complex, obtained from a crystal where GETV macro domain had been co-crystallized with ADP-ribose and subsequently soaked in a solution containing aspartic acid, ADP-ribose had been found in the binding pocket with the distal ribose in the low energy 2′′-*endo* twist conformation and in a position commonly observed in other macro domain/ADP-ribose complexes (pose 1). However, when ADP-ribose and glutamic acid were added to the co-crystallization solution, in the resulting crystal structure the distal ribose (pose 2) was tilted with respect to the one observed in the previously determined ADP-ribose complex and adopted the energetically less favourable *O*-4′′-*endo* envelop configuration. Though, this strained ribose conformation is stabilized by a tighter network of hydrogen bonds contracted with the GETV macro domain when compared to the pose 1 complex structure. Furthermore, in one of the two molecules in the asymmetric unit an acetate molecule, reminiscent of glutamic acid added to the crystallization solution, coordinates the 2′′-OH and 1′′-OH hydroxyl groups, evoking the position of the leaving group after the de-ADP-ribosylation reaction. Apparently, it was the crystallization method deployed, soaking versus co-crystallization with carboxylic acids, which induced the conformational change between pose 1 and pose 2. When GETV macro domain was co-crystallized with ADP-ribose and aspartic acid, the distal ribose was found in two different open conformations, one of them closely resembling the closed-ring ribose of pose 2, the other resulting from a rotation around C4′′ and C3′′, which positioned the aldehyde function close to the α-phosphate, buried in a pocket surrounded by Cys34, Val33, Asn21 and Ala22. Open conformations of an integral ribose in the open conformation bound to macro domains have so far not been observed, but dehydrated open ADP-ribose adducts have been observed in the crystal structures of TARG1^38^ and DraG^32^. Lastly, co-crystallization of GETV macro domain with low concentrations of ADP-ribose and aspartic acid (3 mM each) led to a structure where the distal ribose is covalently attached to Cys34. This covalent thio-hemiacetal adduct is reminiscent of the keto-amine transition states involving a lysine residue described for DraG^32^ and Targ1^38^. It is interesting to notice that an aspartate, Asp184, proposed to be the catalytic acid/base catalyst in human MacroD1^40^, is isosteric to Cys34, as well as Asp102 of human MacroD2^39^ and Glu114 of *T. curvata* PARG^41^, two residues proposed to play crucial roles in catalysis. Noteworthily, DraG, Targ1, MacroD1 and MacroD2 are among the closest structural homologues of GETV macro domain. The above mentioned structural similarities advocate for Cys34 being the catalytic residue in the GETV macro domain, and maybe as well in macro domains of other alphaviruses. Cys-dependent hydrolases found in Nature are mainly represented by Cys-proteases, operating through a catalytic mechanism relying on a Cys-His-Asp catalytic triad. GETV macro domain exhibits no overall structural similarity with any known Cys-protease and the only His and Asp residues found in the vicinity of Cys34, namely His67 and Asp31, though pretty well conserved throughout alphavirus macro domains, are too far apart to form a functional catalytic triad.

The role of the peculiar Ser30 substituting a glycine in the catalytic loop of the GETV macro domain could ultimately not be resolved by this study. The serine residue might contribute to the trapping of ADPr in different poses that were to date not observed in the other alphavirus macro domains having a glycine in position 30. Still, the tight interactions of this residue with the distal ribose observed in the different complex structures described herein plead for a function in substrate binding or eventually a direct role in catalysis in interplay with Cys34.

Altogether the five different conformations of ADP-ribose presented in this study could picture a conformational itinerary (Fig. 5) representing the second part of the de-ADP-ribosylation reaction, leading from the covalent reaction intermediate through different conformational arrangements of the open ring conformations to the final products, represented by pose 2, strained in an energetically unfavourable conformation in the presence of the leaving group, and finally collapsed into the energetically favoured 2′′-*endo* twist conformation (pose 1) as observed in most macro domain/ADP-ribose complexes documented so far. We cannot ascertain whether the covalent Cys34-ADP-ribose adduct portrays the true interaction intermediate, or whether it represents just an artefact standing for an abortive complex. In any case, the conservation in alphavirus macro domain sequences and the proximity of Cys34 to the catalytic centre argues for an important role of this residue in the catalytic mechanism. In this respect, mutational studies, which go beyond the purpose of this report, will be necessary to ascertain the true identity of the catalytic residue of the GETV macro domain.

## Conclusion

In this study, we describe by means of crystallographic structures different poses adopted by a molecule of ADP-ribose in the binding site of GETV macro domain. In addition to the pose of ADP-ribose found in other structures of alphavirus macro domain, this work reveals original features such as the opening of the distal ribose, and its stabilization by Ser30, representing a peculiar GETV specific substitution in the catalytic loop. In addition, we were able to identify a covalent link between ADP-ribose and a cysteine, Cys34, located in the catalytic loop, as well as several poses of ADP-ribose susceptible to provide clues about the catalytic mechanism. Since Cys34 is conserved in alphaviruses, this finding would deserve to be further explored by enzymatic assays and reverse genetics.

## Supplementary information

Supplementary Information.

## Data Availability

The atomic coordinates and structure factors for the structures of GETV macro domains and complexes thereof have been deposited in the Protein Data Bank with accession numbers 6QZU, 6R0F, 6R0G, 6R0P, 6R0R and 6R0T. All the other data are available from the corresponding authors on request.
