# Supplementary material for: Snapshots of ADP-ribose bound to Getah virus macro domain reveal an intriguing choreography
Source: Sci Rep. 2020 Sep 2;10:14422. doi: 10.1038/s41598-020-70870-w (PMC7468284; doi:10.1038/s41598-020-70870-w)
Supplement: Supplementary file 1 — Supplementary Information. [file 41598_2020_70870_MOESM1_ESM.pdf]

Snapshots of ADP-ribose bound to Getah virus macro domain reveal an intriguing choreography.

Ana Sofia Ferreira-Ramos[a,1,2], Gerlind Sulzenbacher[a,2,*], Bruno Canard[a], Bruno Coutard[b,*]

[a] Architecture et Fonction des Macromolécules Biologiques, CNRS, Aix-Marseille Université, 13288 Marseille, France

[b] Unité des Virus Emergents (UVE: Aix Marseille Univ, IRD 190, INSERM 1207, IHU Méditerranée Infection), 13005 Marseille, France.

[1] Present address: Institute of Biochemistry and Molecular Biology, University of Lübeck, Ratzeburger Allee 160, 23562, Lübeck, Germany.

[2] These authors contributed equally to the work

* Corresponding authors: gerlind.sulzenbacher@afmb.univ-mrs.fr (G. Sulzenbacher); bruno.coutard@univ-amu.fr (B. Coutard)

Supplementary Information

Figure S1. Representative electron density maps for ADP-ribose bound to GETV macro domain. GETV macrodomain is represented in cartoon, with β-sheets coloured in slate, α-helices in teal and loops in pink. ADP-ribose is represented in stick-mode, with carbon atoms coloured in grey, oxygens in red, nitrogen atoms in blue and phosphorus atoms in orange. Weighted *Fo-Fc* difference electron density maps, calculated before incorporation of the ligand into the model and contoured at 3.0 σ are shown in green. (**A**) Complex with ADP-ribose in “pose 1”. (**B**) Complex with ADP-ribose in “pose 2”. (**C**) Complex with ADP-ribose in the double open conformation. (**D**) Complex with ADP-ribose in the single open conformation. (**E**) Complex with ADP-ribose covalently bound to Cys34.

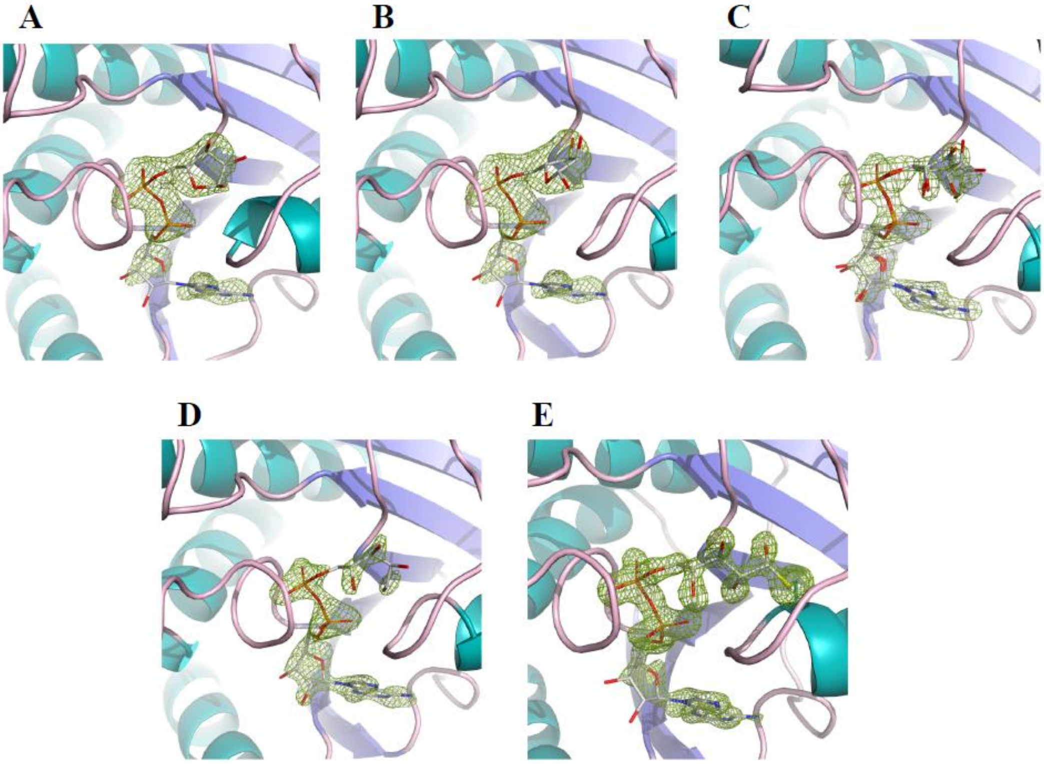
A
B
C
D
E
